# Purification and synergistic antibacterial activity of arginine derived cyclic dipeptides, from *Achromobacter* sp. associated with a rhabditid entomopathogenic nematode against major clinically relevant biofilm forming wound bacteria

**DOI:** 10.3389/fmicb.2015.00876

**Published:** 2015-08-25

**Authors:** Indira Deepa, Sasidharan N. Kumar, Ravikumar S. Sreerag, Vishnu S. Nath, Chellapan Mohandas

**Affiliations:** Division of Crop Protection, Central Tuber Crops Research InstituteThiruvananthapuram, India

**Keywords:** *Achromobacter* sp., arginine, diketopiperazines, antibacterial, wound

## Abstract

Skin and chronic wound infections caused by various pathogenic bacteria are an increasing and urgent health problem worldwide. In the present investigation ethyl acetate extract of an *Achromobacter* sp. associated with a *Rhabditis* entomopathogenic nematode (EPN), displayed promising antibacterial property and was further purified by silica gel column chromatography to get three different cyclic dipeptides (CDPs). Based on the spectral data and Marfey's analyses, the CDPs were identified as cyclo(D-Leu-D-Arg) (1), cyclo(L-Trp-L-Arg) (2), and cyclo(D-Trp-D-Arg) (3), respectively. Three CDPs were active against all the 10 wound associated bacteria tested. The significant antibacterial activity was recorded by CDP 3, and highest activity of 0.5 μg/ml was recorded against *Staphylococcus aureus* and *Pseudomonas aeruginosa*. The synergistic antibacterial activities of CDPs and ampicillin were assessed using the checkerboard microdilution method. The results of the current study recorded that the combined effects of CDPs and ampicillin principally recorded synergistic activity. Interestingly, the combination of CDPs and ampicillin also recorded enhanced inhibition of biofilm formation by bacteria. Moreover, CDPs significantly stimulate the production of IL-10 and IL-4 (anti-inflammatory cytokines) by human peripheral blood mononuclear cells. CDPs do not make any significant effect on the production of pro-inflammatory cytokines like TNF-α. The three CDPs have been studied for their effect on intracellular *S. aureus* in murine macrophages (J774) using 24 h exposure to 0.5X, 1X, and 2X MIC concentrations. Significant decrease in intracellular *S. aureus* burden was recorded by CDPs. CDPs also recorded no cytotoxicity toward FS normal fibroblast, VERO, and L231 normal lung epithelial cell lines. Antimicrobial activity of the arginine containing CDPs against the wound associated bacteria is reported here for the first. Moreover, this is also the first report on the production of CDPs by *Achromobacter* sp. Finally, we conclude that the *Achromobacter* sp. is an incredibly promising source of natural bioactive secondary metabolites especially against wound pathogenic bacteria that may receive significant benefit in the field of human medicine in near future as topical agents.

## Introduction

Hospital-acquired infections remain one of the important causes of morbidity, extended hospital stay and death for many patients worldwide (Sulaiman and Zayed, 2014). The wound and burn represent a susceptible site for many opportunistic microorganisms of endogenous and exogenous origin (Sulaiman and Zayed, 2014). Chronic wounds and burns are one of the worldwide health problems, independent of various socioeconomic and geographic boundaries (Percival et al., 2011). In the United States alone, chronic wounds/burns involve more than 5.5 million patients per year at a cost in excess of 20 billion dollars annually. The occurrence and prevalence of chronic wounds/burns are set to increase among patients irrespective of various disease pathophysiology and age (Percival et al., 2011). The pathogenic microorganisms that are normally isolated from various chronic wounds have included *Staphylococcus aureus, Corynebacterium* spp., *Pseudomonas aeruginosa, Candida albicans*, etc. (Moore et al., 2010; Percival et al., 2011). But, *S. aureus* is the leading causative organism of many serious acute and chronic wound/burns infections in human beings and is one of the major pathogenic microorganisms associated with wound/burn (Dowd et al., 2008; Fazli et al., 2009; Harbarth et al., 2011; Müller et al., 2013). In *S. aureus* strains, methicillin-resistant *S. aureus* (MRSA) have become increasingly common in wounds/burns, and the wide spread of MRSA signifies a solemn health threat to humans and animals around the globe (Müller et al., 2013). However, long-lasting wounds are colonized by many adverse polymicrobial microflora that can increase the risk of an infection development in patients.

The antimicrobial resistance is a growing concern for public health worldwide (Levy and Marshall, 2004). The drug resistant microorganisms of most concerns to wound/burn are often called “superbugs” and these drug resistant microorganisms have the capability to resist various clinical used antibiotics currently (Percival et al., 2011). Antibiotic-resistant bacteria in wounds/burns include MRSA, glycopeptide-resistant enterococci, and multidrug-resistant strains of *Acinetobacter baumannii* and *P. aeruginosa*, which all present a very serious management risk. Subsequently, when infections happen in wounds/burns and the colonizing microorganism frequently grow in biofilms, which are highly resistant to many clinically antibiotics and our ability to cure these infections is very much limited (Kennedy et al., 2010; Percival et al., 2011; Vojtová et al., 2011). In these circumstances, sepsis in burn patients becomes a major concern (Kennedy et al., 2010). Wound/burn associated infections are one of the most common surgical complications in patients that may lead to high levels of morbidity and mortality. Surgical and burn infections are reported as one of the most common form of hospital-acquired infections and reported from both the UK and the USA to occur in more than 40% of patients (Valencia et al., 2004; Percival et al., 2011). Research on hospital infections in India reveals several concerning trends like post-operative wound infection with multidrug-resistant bacteria like MRSA, vancomycin-resistant *S. aureus* (VRSA), carbapenem-resistant *P. aeruginosa*, etc. (Setty et al., 2014). In India, more than 80% of *S. aureus* isolated from hospital-acquired infections were resistant to methicillin and vancomycin (Rao et al., 2013). Moreover, the incidence of MRSA varies from more than 25% in western part of India to 50% in South India [Indian Network for Surveillance of Antimicrobial Resistance (INSAR) Group, 2013]. Industrial development of novel groups of antimicrobial agents has reduced over last 15–20 years, and few pharma companies remain active to find out novel drugs with antibiotic property from natural sources (Müller et al., 2013). So, there is a very urgent need for novel methods or antibiotics to treating various multidrug-resistant bacterial infections are very much essential.

To combat multi-drug resistant wound pathogens, the combination of two or more antibiotics is widely practiced. Moreover, combination of antibiotics can result in synergistic effect to offer improved antimicrobial activity and this also led to the reduction of quantity of individual antibiotic used, which in turn diminish the risk of various unwanted side effects and other treatment costs (Lee et al., 2007; Leibovici et al., 2010; Müller et al., 2013). Moreover, antibiotics in synergistic combination with different mechanism of action diminish the possible risk of drug-resistance arising during chemotherapy (Müller et al., 2013). This is predominantly crucial for patients with chronic wounds/burns where drug therapy is often continued for a very long period.

EPN carrying symbiotic bacteria (mainly *Photorhabdus* or *Xenorhabdus*, respectively) symbolize one of the best eco-friendly strategies to control many insect pests (Lang et al., 2011; Zhu et al., 2011; Salvadori et al., 2012). In natural condition, the active infective juveniles (IJ) of these EPN (Heterorhabditis or Steinernema genera) actively searching for suitable host in the soil, enter through the insect's natural openings like anus, mouth etc., reaching hemocoel where they release the symbiotic bacteria (Salvadori et al., 2012). In hemocoel bacteria multiply vigorously and secrete a large number of chemicals, including various toxin complexes, hydrolytic enzymes, hemolysins, and antimicrobial compounds, which kill the insect host usually within 24–48 h (Ffrench-Constant et al., 2007; Eleftherianos et al., 2010; Salvadori et al., 2012). Due to the presence of diverse antimicrobial compounds, the cadaver remains without putrification for so many days thus providing required nutrients for the nematodes development and reproduction (Salvadori et al., 2012).

During our research on EPN, a novel EPN belonging to the genus *Rhabditis* and subgenus *Oscheius* was isolated from soil samples collected from cassava field, Kollam, Kerala, India (Mohandas et al., 2007). The nematodes can be cultured in *Galleria mellonella* larvae (laboratory reared) and preserved alive for several years in fresh water. A specific symbiotic bacterium were found to be associated with the *Rhabditis* nematode (Mohandas et al., 2007) and can be isolated from the hemolymph of EPN infested *G. mellonella* larvae. Based on molecular characteristics, nematode resembles *Rhabditis (Oscheius)* (Deepa et al., 2010). The bacterial cell free culture filtrate was found to inhibit numerous human and animal pathogenic microorganisms, signifying the presence of many bioactive molecules with antimicrobial property (Mohandas et al., 2007). In this study, we reported the taxonomic study of the symbiotic bacteria, purification, and structure elucidation of three arginine based cyclic dipeptides, its synergistic antimicrobial activity with special reference to major clinically relevant wound bacteria. The current study reports the antimicrobial activity of arginine based cyclic dipeptides for the first time from *Achromobacter* sp.

## Materials and methods

### Chemicals and media

The solvents used for extraction, silica gel column chromatography and High Performance Liquid Chromatography (HPLC) were procured from Merck India Limited, Mumbai, India. Silica gel (230–400 mesh) used for column chromatography and precoated silica gel 60 GF_254_ plates used for Thin Layer Chromatography (TLC) were from Merck Limited, Germany. Media used for microbiological assays were procured from Hi-Media Laboratories Limited, Mumbai, India. All other chemicals and reagents used in the present investigation were of the highest purity. The standard antimicrobial drug ampicillin was procured from Sigma-Aldrich (USA). The Chemsketch Ultra software (Toronto, Canada) was used for drawing the chemical structure of isolated compounds.

### Wound associated microbial targets

The major wound associated bacteria used in the present study are Gram-positive: *Bacillus subtilis* (MTCC 2756), *S. aureus* (MTCC 902), *Staphylococcus epidermidis* (MTCC 435), *Streptococcus faecalis* (MTCC 5383); Gram-negative: *Escherichia coli* (MTCC 2622), *Klebsiella pneumoniae* (MTCC 109), *Proteus mirabilis* (MTCC 425), *P. aeruginosa* (MTCC 2642), *Salmonella typhi* (MTCC 3216), *Proteus vulgaris* (MTCC 742)*, Enterococcus faecium* (MTCC 439). The test pathogens were obtained from Microbial Type Culture Collection Centre (MTCC), Institute of Microbial Technology (IMTECH), Chandigarh, India and maintained on nutrient agar (NA) slants at 4°C.

### Bacterial isolation

The beneficial bacterium was isolated from the haemolymph of *G. mellonella* infected with infective juveniles of *Rhabditis* sp. Dead *G. mellonella* larvae were surface sterilized in 70% alcohol for 10–15 min, flamed for few seconds and allowed to dry in a laminar airflow chamber for 15–20 min. Then larvae were opened with sterile needles and scissors, without damaging the gut portion, and a loop full of oozing haemolymph was streaked onto nutrient agar plates. After 48 h incubation at 30°C, single colonies on the NA plates were selected and aseptically transferred to fresh NA slants.

### Molecular characterization of symbiotic bacterium

#### DNA extraction and 16s rDNA sequencing

For the extraction of genomic DNA, isolated bacteria colonies were collected from the NA plates in 500 μl sterile double distilled water in 1.5 ml microtubes. Cells were disrupted by heating at 100°C for 10 min and then centrifuged at 10,000 g for 2 min and the supernatants were deproteinized by adding 200 μl of chloroform: isoamylic acid (24:1 v/v) followed by centrifugation for 10 min at 10,000 g. The aqueous portion containing DNA were transferred to fresh 1.5 ml microtubes and stored at −20°C for further studies. Previously reported primers were used for amplify the 16S rDNA region (Kim et al., 2009; Salvadori et al., 2012).

PCR amplification was done according to the method of Saiki et al. (1988), with some minor changes. The standard 50 μl PCR mixtures contained 5 × reaction buffer (10 μl), dNTP mixture (1 μl, 2.5 mM each), 5 μM forward primer (2 μl), 5 μM reverse primer (2 μl), Taq polymerase (0.25 μl; 5 U/μl), template DNA (3 μl), and sterile distilled water (31.75 μl). The controls tubes contained all components used for PCR except template DNA. After that, the PCR mixtures were incubated in a PCR thermal cycler for a series of 35-cycle amplification. After initial denaturation (94°C for 3 min), each cycle included denaturation at 94°C for 45 s, annealing at 62°C for 45 s, and extension at 72°C for 2 min. After that the final extension was carried out at 72°C for 7 min. The amplified products were separated on agarose gel (1%) containing ethidium bromide (0.5 μg/ml) and were viewed under UV light using Gel documentation system (Bio-Rad). Final PCR products were further purified with Illustra™ GFX™ PCR DNA and Gel Band Purification Kit (GE Healthcare, UK) following the manufacturer's instructions. DNA Sequencing was carried out at the DNA fingerprinting unit of Rajiv Gandhi Center for Biotechnology (RGCB), Trivandrum, Kerala, India using a PCR thermal cycler (GeneAmp PCR System 9700, Applied Biosystems) using the BigDye Terminator v3.1 Cycle sequencing Kit (Applied Biosystems, USA) following manufacturers protocol. The genes were then finally aligned to the corresponding sequences available in the NCBI nucleotide database by nBLAST.

#### Phylogenetic analyses of the sequences

Mega (Molecular Evolutionary Genetic Analysis) software version 5.0 (Tamura et al., 2007) was used to analyze the evolutionary distances following Kimura-2 parameter (Kimura, 1980). The phylogenetic tree was created by the neighbor-joining method, and bootstrap analysis was used to assess the tree topology by performing at least 1000 re-samplings (Saitou and Nei, 1987). The taxonomic status of the present strain was established based on 16S rDNA homology.

#### Fermentation and extraction of secondary metabolites

The bacterial fermentation for antibacterial secondary metabolite production was carried out using a modified Tryptic soya broth (TSB) (original TSB media supplemented with 1% beef extract) as reported earlier (Kumar et al., 2014a). A total of 25 L of bacterial cell-free culture filtrate were produced and neutralized with the concentrated hydrochloric acid and extracted with ethyl acetate (equal volume) thrice, dried over anhydrous sodium sulfate, and concentrated at 42°C using a Buchi rotary flash evaporator (Kumar et al., 2014a).

#### Fractionation of the crude ethyl acetate extract

The fractionation of ethyl acetate extract was done according to our previously reported method with minor modification in the combination of solvent system (Kumar et al., 2014a). Heat activated silica gel (230–400 mesh) was packed into a glass column (600 × 30 mm) using n-hexane and eluted sequentially with 500 ml of n-hexane (100%), 500 ml of linear gradient hexane: dichloromethane (v/v, 90:10–10:90), 500 ml of dichloromethane (100%), 500 ml of linear gradient dichloromethane: ethyl acetate (v/v, 95:5–5:95), 500 ml of ethyl acetate (100%), and finally with 500 ml of acetone (100%). Five fractions (100 ml each) were collected from each solvent combination and concentrated by using the Buchi rotary evaporator at 40°C (Kumar et al., 2014a).

#### Thin-layer chromatography (TLC)

TLC profile of the compounds was carried out on silica gel plates (Merck 60, F_254_) using ethyl acetate and acetone (90:10 v/v). The compounds were located by exposing the TLC plate to iodine fumes.

#### Reverse phase-high pressure liquid chromatography (RP-HPLC)

The final purity of the compounds were analyzed by an LC-10AT liquid chromatography (Shimadzu, Singapore) equipped with a C-18 column (5 μm, 4.6 × 250 mm). Samples of 20 μl were injected to a column and eluted using the following gradient program: solvent A (water), solvent B (MeCN); 0 min 50% B, 15 min 75% B, 25 min 100% B; UV detection at 210 nm with a flow rate of 1 ml/min.

#### Structure elucidation of pure compounds

^1^H NMR and ^13^C NMR spectra were acquired using a Bruker DRX 500 NMR spectrometer (Bruker, Rheinstetten, Germany) at 20°C and Chemical shifts are reported relative to the corresponding reference solvent (DMSO-d6: ^1^H δ 2.50 and ^13^C δ 39.52). Thermo Scientific Exactive Mass Spectrometer (Thermo Fisher Scientific, Bremen, Germany) with an electrospray ionization mode was used to record HRESIMS. Optical rotations were measured on a Rudolph Research Autopol III digital polarimetric apparatus (Hackettstown, NJ, USA). Melting points (m.p.) were determined on a Mettler Toledo DSC 822e apparatus (Mettler-Toledo, Schwerzenbach, Switzerland).

#### Marfey's analysis (acid hydrolysis) of pure compounds

Compounds (1.5 mg) each was dissolved in 0.1 ml 6 mol/L HCl and hydrolyzed at 110°C for 20 h. After drying, the hydrolysate was dissolved in distilled H_2_O and was then placed in a 1 ml reaction tube and treated with a 2% solution of FDAA (200 μl) in acetone followed by 1.0 mol/L NaHCO_3_ (40 μl). This reaction mixture was heated at 49°C for 1 h, cooled to room temperature, and then acidified with 2.0 mol/L HCl (20 μl). In a similar way, standard D- and L-amino acids were derivatized separately. RP-HPLC of the hydrolysate was done in a Shimadzu LC-20AD, C-18 column; (5 μm, 4.6 × 250 mm) with a flow rate of 1.0 ml/min at 30°C using an eluent system consisting of 0.2% aqueous TFA (eluent A) and MeCN (eluent B). A linear gradient from 25% eluent B (0 min) to 60% eluent B (40 min) and to 100% eluent B (45 min) was applied. Absorbance was recorded at 340 nm (Marfey, 1984).

### Antibacterial activity of compounds against wound pathogens

#### Determination of minimal inhibitory concentration (MIC) and minimal bactericidal concentration (MBC)

The MIC value of the compounds against the test wound associated pathogens was determined by microdilution method as suggested by Clinical and Laboratory Standards Institute (CLSI, 2012). The compounds were transferred to a 96 well microplate in order to get twofold serial dilutions of the test compounds and antibiotics. After that, 20 μl inoculum containing 5 × 10^5^ CFU/ml of corresponding test bacteria was added to each well, and the microplates were incubated at 37°C for 24 h. Wells without test pathogens were used as sterility control, and the positive controls encompassed of test bacteria without the test compounds. After incubation, bacterial growth was indicated by the presence of turbidity, which was confirmed by adding 20 μl of resazurin (Sigma-Aldrich, USA) and incubated for 1 h at 37°C. The MIC was defined as the lowest concentration of the test compound/antibiotics that was able to inhibit the bacterial growth. Ampicillin was used as a standard antimicrobial agent (Silva et al., 2013).

The serial dilution method was also used to establish the MBC values. For this, 20 μl of the microbial culture were withdrawn from wells with concentrations of compounds equal to, or higher than, the MIC, serially diluted, plated on nutrient agar plates, and incubated at 37°C for 24 h and colonies were counted after incubation (Silva et al., 2013). The MBC was defined as the lowest concentration of compound/antibiotics at which the entire test bacteria have been killed (99.99% reduction). The experiments were performed for three times.

#### Disc diffusion test to check antibacterial susceptibility

Antibacterial susceptibility testing of the compounds was performed by standard disk diffusion assay according to CLSI protocol (CLSI, 2009). The inoculum was prepared by picking the test bacteria from plates with a sterile inoculating loop and suspended in sterile normal saline (0.85%). The density of the suspension to be inoculated was determined by comparison with McFarland 0.5 standard. After that, the test bacteria was swabbed uniformly over the Mueller-Hinton agar (MHA) plates, and filter paper disc (6 mm, Hi-media) incorporated with MIC concentration of the test compounds are placed in the agar medium. After that, the plates were incubated for 18 h at 37°C and the diameters of zones of inhibition around the discs were measured in millimeter (mm). The experiments were repeated for three times.

#### Synergistic activity of the test compounds and antimicrobial drug against wound pathogens

The synergistic antimicrobial activity of the test compounds and antimicrobial drug (ampicillin) was determined by a checkerboard assay according to the modified method of Silva et al. (2013), with the CLSI microdilution method. The test compounds and ampicillin were combined in concentrations lower than their individual MIC values by serial dilution in 96 well microplates. The bacterial test inoculum (20 μl) containing 5 × 10^5^ CFU/ml was added to each well, and the microplates were incubated for 24 h at 37°C. The bacterial growth was confirmed by adding resazurin (20 μl) and incubated for 1 h at 37°C (Lee et al., 2012). Triplicate set of experiments were performed.

To assess the interaction of combinations of test compounds and ampicillin, the results obtained were further analyzed using the fractional inhibitory concentration (FIC) index. The interaction of two compounds was regulated as synergy, indifferent or antagonistic on the basis of the FIC index values.

The FIC-values were calculated as follows:

FIC of compound A (test compounds) (FIC_A_) = (MIC of compound A in combination)/(MIC of compound A alone)
FIC of compound B (ampicillin) (FIC_B_) = (MIC of compound B in combination)/(MIC of compound B alone)
The sum of fractional inhibitory concentration indices (FICI) of two compounds (A and B) in the combination was calculated as follows:
FIC_A_ + FIC_B_ = FICI.
The interaction between compounds and antibiotics was considered to be synergistic when FICI ≤0.5, indifferent at 0.5 < FICI < 4, and antagonistic when FICI ≥4 (Cordeiro et al., 2003).

The fractional bactericidal concentration (FBC) is calculated in the same way described above by substituting the MIC values with the MBC values.

### Inhibition of bacterial biofilm formation by the combination of test compounds and ampicillin

The test compounds and ampicillin alone and in combinations were tested for their potential to prevent biofilm formation in the wound associated bacteria. The test bacteria were added to the growth medium at the time of inoculation, and the bacterial cells were allowed to form the biofilm. An aliquot of twofold serial dilutions (100 μl) was prepared in the 96-well microtitre plate containing TSBGlc as growth medium, with the MIC concentrations of test compounds and ampicillin alone and in combination. The test bacterial suspensions (20 μl; 5 × 10^5^ CFU/ml) were then added to the plate. TSBGlc containing 0.2% DMSO was employed as a negative control. Non-treated control TSBGlc without the test compounds and ampicillin and the medium with each concentration of the test compounds was used as the blank control (Chusri et al., 2012). After incubation for 24 h at 37°C, the impact of the combinations on the growth of test bacteria was evaluated using the microplate (ELISA) reader (Bio-rad, USA) at an optical density of 600 nm. The biofilm formation of test bacteria in the presence of the combination of test compounds and ampicillin was recorded using the MTT (3-(4,5-dimethyl-2-thiazolyl)-2,5-diphenyl-2H-tetrazolium-bromide) assay.

MTT (Sigma-Aldrich, USA) reduction assay according to the method previously reported by Tang et al. (2011) was used to quantify the biofilm forming capability of the test pathogens. An aliquot (200 μl) of MTT solution (0.2 mg/ml) was added to each of the prewashed wells, and the plate was again incubated at 37°C for 3 h in the dark. After 3 h, MTT solution was replaced by adding DMSO (200 μl). Bacterial pathogens with an active electron transport system will easily reduce the tetrazolium salt to a water-insoluble purple formazan product (Chusri et al., 2012). The color intensity was determined by an ELISA microplate reader (Bio-rad, USA) at 482 nm. The absorbance values for the negative control were then subtracted from the tested wells to eliminate false results due to background interference. Triplicate set of experiments were performed (Chusri et al., 2012).

### Immunomodulatory property of test compounds

#### Cytokine release measurement

Peripheral blood mononuclear (PBM) cells were isolated from the heparinized venous blood of healthy donors by density gradient centrifugation using Histopaque 1077. The PBM cells were then washed three times with RPMI 1640 medium and finally resuspended in the RPMI 1640 containing 10% fetal bovine serum (FBS), 2 mM L-glutamine, penicillin G (100 IU/ml), and streptomycin (100 μg/ml). Trypan blue staining was used to determine the cell number and viability. Freshly isolated PBM cells were grown in 96 well plates at an initial density of 2 × 10^5^ cells/well and incubated with test compounds (0.1, 1, and 10 μg/ml). The control well contains medium alone. The plates marked as Con A in **Figure 5** consisted of cells treated in the similar way but in the presence of standard concanavalin A (2.5 μg/ml). Cells were incubated for 36 h at 37°C in a humidified atmosphere of 5% CO_2_–95% air. After incubation cells were centrifuged, supernatants collected and kept at −20°C for further analysis (Mechkarska et al., 2013).

Cytokine concentrations in the supernatants (diluted 1:4) were measured by ELISA reader (Bio-rad, USA) by OptEIA assay kits from BD Biosciences (San Diego, USA) according to manufacturer's protocols: tumor necrosis factor-α (TNF-α), IL-4, and IL-10. Triplicate set of experiments were performed (Mechkarska et al., 2013).

#### Effect of test compounds on murine macrophage J774 cells treated with *S. aureus*

To check the effect of test compounds on murine macrophage cell line (J774), the murine macrophage was cultured in RPMI 1640 medium supplemented with L-glutamine and FBS. For bacterial (*S. aureus*) infection studies, J774 cells were transferred to 12 well tissue culture plate in 1 ml volumes at a density of 2 × 10^5^ in the presence of 10% FBS. After overnight incubation, the medium was replaced with fresh medium containing 1% FBS to arrest macrophage cell division while maintaining cell viability. After 24 h, the macrophage monolayer was counted with an ocular micrometer for the total number of cells per well to determine the infection ratio. Phagocytosis was started at a *S. aureus*-macrophage ratio of 4:1, and after removal of unphagocytosed and adherent *S. aureus* cells, the inoculum was typically 0.5–2 × 10^6^ bacteria per mg of cell protein, as for J774 macrophages. The cells were infected for 4 h and after that, non-phagocytosed *S. aureus* cells were washed from the monolayers, and fresh medium was added. 0.5X, 1X, and 2X MIC concentrations of test compounds were added and incubated for 24 h. After incubation, the macrophages were lysed with sodium dodecyl sulfate (SDS), treated with DNAase, diluted and plated onto NA plates to determine the *S. aureus* cell number or colony forming units (CFU). The experiment was repeated thrice to confirm the observations. A cytotoxicity control plate assay (MTT) was also conducted in parallel using uninfected macrophages to confirm that concentrations utilized for testing were not toxic to the macrophages (Papadopoulou et al., 2014).

#### Cytotoxicity of pure compounds

The MTT assay was used to check whether the isolated compounds are cytotoxic to the normal cells or not. FS normal fibroblast, VERO (African green monkey kidney), and L231 normal lung epithelial cell lines were used for the present study. Briefly, normal cells (5 × 10^3^ per well) were seeded in 0.2 ml of the medium (DMEM with 10% PBS) on 96 well plates, treated with test compounds (5–100 μg/ml) for 72 h. After incubation cytotoxicity was measured by removing the compounds containing media from the well and 25 μl of MTT solution (5 mg/ml in PBS) and 75 μl of complete medium were added to wells (treated and control) and incubated for 2 h. After incubation, MTT lysis buffer was added to the wells (0.1 ml/well) and again incubated for another 4 h at 37°C. After that, the OD at 570 nm was measured using an ELISA plate reader (Bio-Rad, USA). The relative cell viability (%) was calculated (A _570_ of treated well)/(A _570_ of untreated well) × 100. Triplicate set of experiments were performed (Kumar et al., 2014b).

#### Statistical analysis

All the statistical analyses were performed with SPSS (Version 17.0; SPSS, Inc., Chicago, IL, USA). In MIC, MBC, and FICI determinations, when the results were different in both experiments, we made another test for the final result. One-Way ANOVA-Duncan's multiple range test was used and the statistical significance was defined as *p* < 0.05.

## Results

### Molecular identification of bacterium

**Figure 1:**
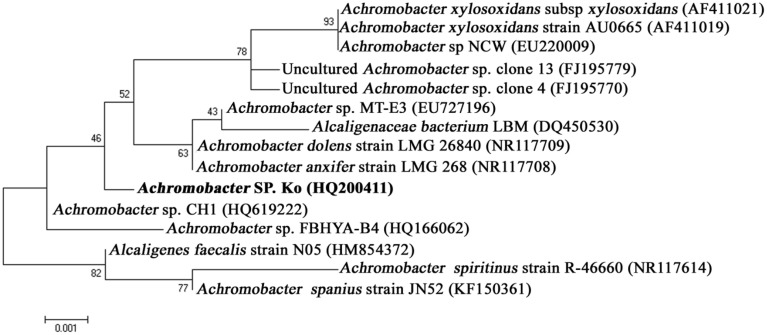
**Phylogenic relationships of *Achromobacter* sp. strain isolated from *Rhabditis* (Oscheius) sp. and known bacterial relatives based on 16S rDNA gene sequences (neighbor-joining method)**.

The bacterium (*Achromobacter* sp.) was identified based on 16S rDNA gene sequencing. PCR amplification yielded ~1500 bp amplicon. BLAST analysis recorded 99% similarity to *Achromobacter* sp. sequence available in the NCBI Genbank database and thus, the test bacterium was identified as *Achromobacter* sp. The 16S rDNA gene sequence data have been deposited in the NCBI nucleotide database (Ac. No. HQ200410). The phylogenetic tree clearly portrayed the relationships of the isolates used in the analysis. Thus, the *Achromobacter* sp. was successfully grouped along with other *Achromobacter* sp. isolates obtained from the NCBI GenBank database confirming the complete authenticity of our strain (Figure 1). This *Achromobacter* sp. was presently deposited in culture collection center of IMTECH (Institute of Microbial Technology, Chandigarh, India).

### Isolation, purification, and characterization of bioactive compounds

**Figure 2:**
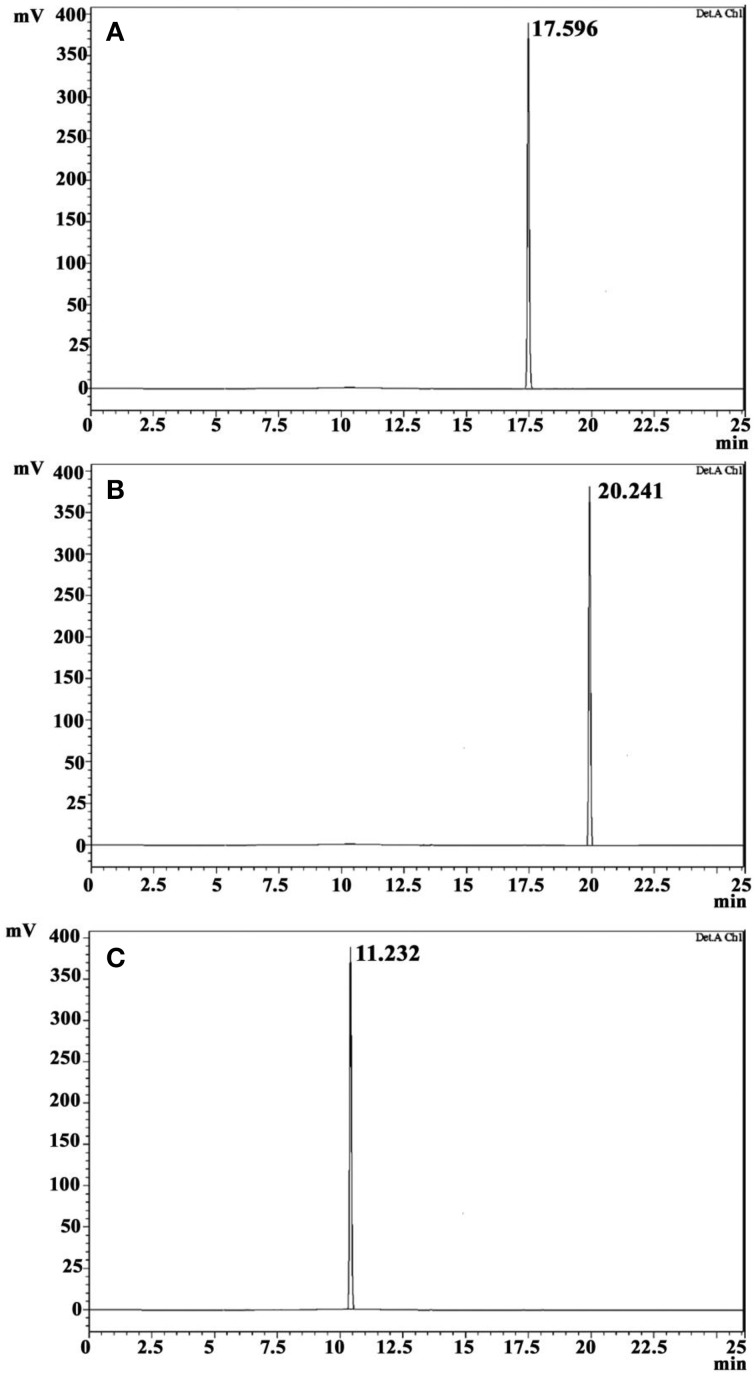
**HPLC profile of CDPs on a reversed-phase C18 HPLC column**. Cyclo(D-Leu-D-Arg) **(A)**, Cyclo(L-Trp-L-Arg) **(B)**, and Cyclo(D-Trp-D-Arg) **(C)**.

The crude ethyl acetate extract recorded promising antibacterial activity against *S. aureus* (indicator microorganism). Column chromatographic purification of ethyl acetate extract yielded three compounds. The eluted solvent system in column chromatography and yield of each compound were revealed in Table 1. The three compounds were further purified by crystallization using hexane and benzene to yield white crystals. Antibacterial activity of these crystals was again confirmed by testing against *S. aureus*. TLC profile of these purified crystal compounds recorded single spots, and *R*_F_-value is presented in Table 1. In HPLC analysis, the compounds were eluted as single peaks, which confirm its purity (Figure 2). The purity of the compounds recorded more than 98%, according to the peak area from the chromatogram.

**Table 1:** **Information regarding the isolated compounds**.

**S.no**.	**Compounds**	**Column solvent**	**Yield (mg)**	**Melting point (°C)**	**Optical rotation (c, 0.02, MeOH)**
1	Cyclo(D-Leu-D-Arg)	30% DCM in hexane	19	202.51−205.55	[a]_D_ − 118
2	Cyclo(L-Trp-L-Arg)	75% DCM in hexane	16	265.1−267.34	[a]_D_ + 145
3	Cyclo(D-Trp-D-Arg)	15% ethyl acetate in DCM	21	262.23−265.58	[a]_D_ − 167

### Identification of cyclic dipeptides as antimicrobial compounds

**Figure 3:**
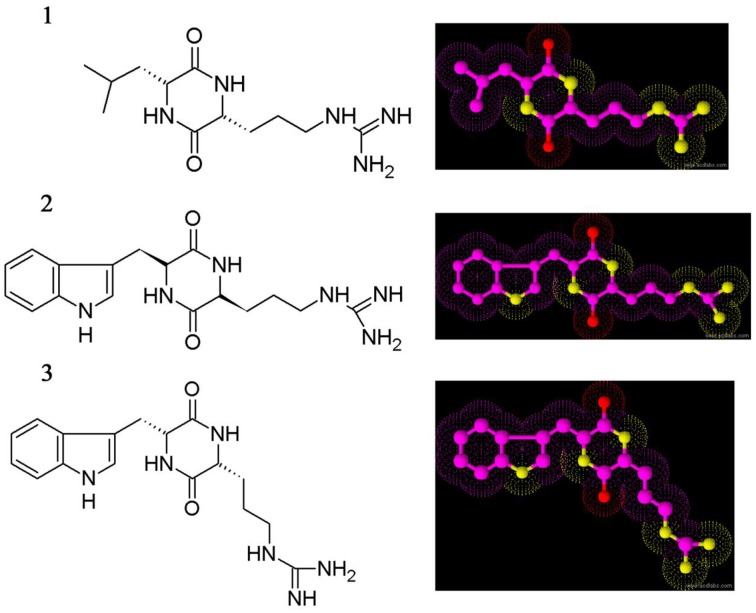
**Chemical structure of cyclic dipeptides**. Cyclo(D-Leu-D-Arg) (1), Cyclo(L-Trp-L-Arg) (2), and Cyclo(D-Trp-D-Arg) (3).

The three crystal compounds were subjected to various spectroscopic analyses for elucidating the chemical structure, i.e., UV, HR-MS, and NMR (^1^H and ^13^C NMR). Based on spectral data the compounds were identified as three different cyclic dipeptides (CDPs) or diketopiperazines. The CDPs identified are cyclo(D-Leu-D-Arg) (1), cyclo(L-Trp-L-Arg) (2), and cyclo(D-Trp-D-Arg) (3), respectively (Figure 3).

**CDP 1: Cyclo(D-Leu-D-Arg) (1-{3-[(2*R*,5*R*)-5-(2-methylpropyl)-3,6-dioxopiperazin-2-yl]propyl}guanidine):** ^1^H NMR (DMSO-d6, 500 MHz) δ 7.91 (1H, br s, Arg-NH), δ 4.13(1H, dd, *J* = 8.2, 8.1 Hz, Arg-H2), δ 4.04 (1H, dd, *J* = 6.8, 5.7 Hz, Leu-H2), δ 3.47 (1H, m, Arg-H5), δ 3.26 (1H, m, Arg-H5), δ 2.18 (1H, m, Arg-H3), δ 1.99 (1H, m, Arg-H3), δ 1.94 (2H, m, Leu-H4), δ 1.75 (2H, m, Arg-H4), δ 1.75 (1H, m, Leu-H3), δ 1.32 (1H, m, Leu-H3), δ 0.83 (3H, d, *J* = 7.0 Hz, Leu-H5), δ 0.81 (3H, d, *J* = 7.0 Hz, Leu-H50); ^13^C NMR (DMSO-d6, 125 MHz) 170.1, 167.4, 58.7, 53.9, 45.9, 38.1, 28.3, 24.1, 24.0, 23.1, and 22.9. The molecular formula of this compound was determined to be C_12_H_24_O_2_N_5_ by HR-ESI-MS at m/z 270.22351 [M+H].

**CDP 2: Cyclo(L-Trp-L-Arg) (1-{3-[(2*S*,5*S*)-5-(1*H*-indol-3-ylmethyl)-3,6-dioxopiperazin-2-yl]propyl}guanidine):** ^1^H NMR (DMSO-*d*6, 500 MHz) 10.77d (d, *J* = 2.3), 8.17 (d, *J* = 2.4), 8.11 (d, *J* = 2.3), 7.69 (dd, *J* = 8.0, 1.0), 7.41 (d, *J* = 8.1), 7.28 d (t, *J* = 5.7), 7.15 (ddd, *J* = 8.0, 7.0, 1.1), 7.12 (s), 7.01 (ddd, *J* = 8.0, 7.0, 1.0), 4.37 (ddd, *J* = 4.8, 3.7, 1.3), 3.74 (ddd, *J* = 7.7, 5.2, 1.5), 3.51 (dd, *J* = 14.8, 3.6), 3.19 (dd, *J* = 14.7, 4.6), 2.72 (d, *J* = 7.0, 2.0), 0.81 (m), 0.79 (m), 0.54 (m). ^13^C NMR (DMSO-d6, 125 MHz) 169.81, 169.28, 158.54, 137.85, 129.41, 126.17, 122.44, 120.35, 120.29, 112.32, 109.60, 57.21, 55.24, 41.68, 32.16, 30.52, and 24.59. The molecular formula of this compound was determined to be C_17_H_23_O_2_N_6_ by HR-ESI-MS at m/z 343.39558 [M+H].

**CDP 3: Cyclo(D-Trp-D-Arg) (1-{3-[(2*R*,5*R*)-5-(1*H*-indol-3-ylmethyl)-3,6-dioxopiperazin-2-yl]propyl}guanidine):** ^1^H NMR (DMSO-*d*6, 500 MHz) 10.91d (d, *J* = 2.3), 8.21 (d, *J* = 2.4), 8.24 (d, *J* = 2.3), 7.77 (dd, *J* = 8.0, 1.0), 7.43 (d, *J* = 8.1), 7.34 d (t, *J* = 5.7), 7.21 (ddd, *J* = 8.0, 7.0, 1.1), 7.22 (s), 7.01 (ddd, *J* = 8.0, 7.0, 1.0), 4.43 (ddd, *J* = 4.8, 3.7, 1.3), 3.88 (ddd, *J* = 7.7, 5.2, 1.5), 3.61 (dd, *J* = 14.8, 3.6), 3.33 (dd, *J* = 14.7, 4.6), 2.77 (d, *J* = 7.0, 2.0), 0.88 (m), 0.79 (m), 0.61 (m). ^13^C NMR (DMSO-d6, 125 MHz) 170.11, 170.01, 158.79, 138.22, 130.28, 127.17, 123.14, 121.38, 121.32, 113.28, 109.66, 59.01, 54.67, 41.99, 32.86, 31.48, and 25.5. The molecular formula of this compound was determined to be C_17_H_23_O_2_N_6_ by HR-ESI-MS at m/z 343.37431 [M+H].

### Absolute configuration determination of cyclic dipeptides

The advanced Marfey's analysis was effectively employed for determining the absolute configuration of CDPs. Regarding the absolute configuration of compounds, the CDP 1 and 3 contain D-amino acids, whereas CDP 2 contains L-amino acids (Data not shown). The three derivatives obtained by the acid hydrolysis of the CDPs were compared with the HPLC retention times of the derivatized standard D and L-amino acids.

### Antibacterial activity of cyclic dipeptides

The pure CDPs were tested for antibacterial activity against 10 wound associated bacterial pathogens using CLSI protocol. MIC and MBC values of CDPs were recorded and are presented in Table 2. The test pathogen that exhibited the highest sensitivity toward CDP 1 was *B. subtilis*. CDP 2 was active against all the test bacteria only at higher concentration and best activity of this compound was recorded against *S. epidermidis* (32 μg/ml), followed by *S. aureus* (64 μg/ml). Interestingly, CDP 3 recorded good activity against all test pathogens in impressively low concentration, and best activity was recorded against *S. aureus* and *P. aeruginosa* (0.5 μg/ml). The activity of the test compounds was better than the ampicillin, standard antimicrobial drug. The disc diffusion assay of the CDPs against test bacteria was shown in Table 3.

**Table 2:** **MIC and MBC of cyclic dipeptides against wound associated bacterial pathogens**.

**Test bacteria**	**MIC (μg/ml)**							
	**CDP 1**		**CDP 2**		**CDP 3**		**Ampicillin**	
	**MIC**	**MBC**	**MIC**	**MBC**	**MIC**	**MBC**	**MIC**	**MBC**
*B. subtilis*	8	16	125	125	4	8	8	16
*S. aureus*	16	32	64	64	0.5	0.5	4	4
*S. epidermidis*	16	16	32	64	8	16	4	8
*S. faecalis*	32	64	500	1000	4	8	8	16
*E. faecium*	16	32	250	250	2	4	2	4
*P. aeruginosa*	125	250	250	500	0.5	0.5	2	2
*P. vulgaris*	500	500	1000	1000	4	8	2	4
*P. mirabilis*	125	125	1000	2000	64	128	4	8
*K. pneumonia*	250	500	125	250	2	4	4	8
*S. typhi*	64	125	125	250	32	64	8	16

*No of MIC up to 1000 μg/ml*.

**Table 3:** **Antimicrobial activity of cyclic dipeptides against wound associated bacteria**.

**Test bacteria**	**Inhibition zone (dia. in mm)**			
	**CDP 1**	**CDP 2**	**CDP 3**	**Ampicillin**
*B. subtilis*	18 ± 1	17 ± 0	24 ± 0	26 ± 0
*S. aureus*	20 ± 1	18 ± 0.57	35 ± 0.57	28 ± 1
*S. epidermidis*	21 ± 1.52	15 ± 0	27 ± 1.15	30 ± 1.15
*S. faecalis*	17 ± 1	15 ± 0.57	30 ± 1.15	29 ± 0
*E. faecium*	23	12 ± 0.57	31 ± 1.15	26 ± 1
*P. aeruginosa*	21	11 ± 0	34 ± 1	25 ± 0.57
*P. vulgaris*	20	13 ± 0.57	29 ± 1.73	30 ± 0
*P. mirabilis*	16 ± 0	13 ± 0.57	30 ± 0	24 ± 1.52
*K. pneumonia*	18 ± 1.2	10 ± 0	27	27 ± 0.57
*S. typhi*	21	9 ± 1.2	23 ± 1	30 ± 1.52

*Values represent mean of three replications*.

### Cyclic dipeptides synergistically enhance the activity of ampicillin against wound pathogens

The combined activities of CDPs with ampicillin from the *in vitro* checkerboard assay against wound associated bacteria are shown in Table 4. FIC, FBC, FIC index, FBC index and interpretations for the activities of CDPs, and ampicillin against the test bacteria predominantly recorded the synergistic interaction, i.e., significant enhancement in the bioactivity. But CDP 1 with ampicillin against *P. aeruginosa* and CDP 2 with ampicillin against *K. pneumonia* recorded additive. Antagonism and indifference were not recorded for the combinations. When CDP 3 was combined with ampicillin for the inhibition of *P. aeruginosa*, an important synergistic effect (FIC = 0.09) was observed and the MIC values of CDP 3 and ampicillin were reduced to more than five times below their individual MIC values, respectively (Table 4). From the checkerboard assay, it is clearly evident that the CDPs enhance the activity of ampicillin against wound bacterial pathogen tested and in most combinations the MIC level has reduced many folds. This data clearly indicated that the combination is more effective than the individual compounds.

**Table 4:** **Synergistic effects of the cyclic dipeptides with ampicillin against wound associated bacteria**.

**Test bacteria**	**Agent**	**MIC/MBC (μg/ml)**		**FIC/FFC**	**FICI^b^/FFCI^c^**	**Outcome**
		**Alone**	**Combination^a^**			
*B. subtilis*	CDP 1	8/16	1/2	0.13∕0.13	0.16∕0.16	Synergistic/synergistic
	Ampicillin	8/16	0.25/0.5	0.03∕0.03		
	CDP 2	125/125	16/16	0.13∕0.13	0.26∕0.26	Synergistic/synergistic
	Ampicillin	8/16	1/2	0.13∕0.13		
	CDP 3	4/8	1/1	0.25∕0.06	0.31∕0.12	Synergistic/synergistic
	Ampicillin	8/16	0.25/0.5	0.06∕0.06		
*S. aureus*	CDP 1	16/32	2/4	0.13∕0.13	0.26∕0.26	Synergistic/synergistic
	Ampicillin	4/4	0.5/0.5	0.13∕0.13		
	CDP 2	64/64	8/16	0.13∕0.25	0.38∕0.5	Synergistic/synergistic
	Ampicillin	4/4	1/1	0.25∕0.25		
	CDP 3	0.5/0.5	0.03/0.06	0.06∕0.12	0.18∕0.37	Synergistic/synergistic
	Ampicillin	4/4	0.5/1	0.12∕0.25		
*S. epidermidis*	CDP 1	16/16	1/1	0.06∕0.06	0.19∕0.12	Synergistic/synergistic
	Ampicillin	4/8	0.5/0.5	0.13∕0.06		
	CDP 2	32/64	4/4	0.13∕0.06	0.26∕0.19	Synergistic/synergistic
	Ampicillin	4/8	0.5/1	0.13∕0.13		
	CDP 3	8/16	1/1	0.13∕0.06	0.25∕0.12	Synergistic/synergistic
	Ampicillin	4/8	0.24/0.24	0.12∕0.06		
*S. faecalis*	CDP 1	32/64	4/8	0.13∕0.13	0.19∕0.19	Synergistic/synergistic
	Ampicillin	8/16	1/2	0.06∕0.06		
	CDP 2	500/1000	32/32	0.06∕0.06	0.09∕0.12	Synergistic/synergistic
	Ampicillin	8/16	4/8	0.03∕0.06		
	CDP 3	4/8	1/1	0.13∕0.06	0.15∕0.08	Synergistic/synergistic
	Ampicillin	8/16	0.12/0.25	0.02∕0.02		
*E. faecium*	CDP 1	16/32	4/8	0.02∕0.02	0.14∕0.15	Synergistic/synergistic
	Ampicillin	2/4	0.25/0.5	0.12∕0.13		
	CDP 2	250/250	32/64	0.13∕0.26	0.38∕0.51	Synergistic/additive
	Ampicillin	2/4	0.5/1	0.25∕0.25		
	CDP 3	2/4	0.5/1	0.25∕0.25	0.31∕0.31	Synergistic/synergistic
	Ampicillin	2/4	0.12/0.25	0.06∕0.06		
*P. aeruginosa*	CDP 1	64/125	16/16	0.25∕0.13	0.31∕0.19	Synergistic/synergistic
	Ampicillin	8/16	0.5/1	0.06∕0.06		
	CDP 2	125/250	8/16	0.06∕0.06	0.19∕0.19	Synergistic/synergistic
	Ampicillin	8/16	1/2	0.13∕0.13		
	CDP 3	0.5/1	0.03/0.03	0.06∕0.03	0.09∕0.06	Synergistic/synergistic
	Ampicillin	8/16	0.25/0.5	0.03∕0.03		
*P. vulgaris*	CDP 1	500/500	64/125	0.13∕0.25	0.38∕0.5	Synergistic/synergistic
	Ampicillin	2/4	0.5/1	0.25∕0.25		
	CDP 2	1000/1000	125/125	0.13∕0.13	0.38∕0.38	Synergistic/synergistic
	Ampicillin	2/4	0.5/1	0.25∕0.25		
	CDP 3	4/8	0.24/0.5	0.06∕0.06	0.07∕0.07	Synergistic/synergistic
	Ampicillin	2/4	0.03/0.06	0.01∕0.01		
*P. mirabilis*	CDP 1	125/125	16/16	0.13∕0.13	0.19∕0.19	Synergistic/synergistic
	Ampicillin	4/8	0.25/0.5	0.06∕0.06		
	CDP 2	1000/2000	125/250	0.13∕0.13	0.38∕0.26	Synergistic/synergistic
	Ampicillin	4/8	1/1	0.25∕0.13		
	CDP 3	64/125	16/32	0.25∕0.25	0.38∕0.38	Synergistic/synergistic
	Ampicillin	4/8	0.5/1	0.13∕0.13		
*K. pneumonia*	CDP 1	250/500	32/64	0.13∕0.13	0.26∕0.26	Synergistic/synergistic
	Ampicillin	4/8	0.5/1	0.13∕0.13		
	CDP 2	125/250	32/64	0.26∕0.26	0.51∕0.51	Additive/additive
	Ampicillin	4/8	1/2	0.25∕0.25		
	CDP 3	2/4	0.12/0.24	0.06∕0.06	0.12∕0.12	Synergistic/synergistic
	Ampicillin	4/8	0.25/0.5	0.06∕0.06		
*S. typhi*	CDP 1	125/250	32/64	0.26∕0.26	0.51∕0.51	Additive/additive
	Ampicillin	2/2	0.5/0.5	0.25∕0.25		
	CDP 2	250/500	32/32	0.12∕0.06	0.37∕0.31	Synergistic/synergistic
	Ampicillin	2/2	0.5/0.5	0.25∕0.25		
	CDP 3	32/64	4/8	0.12∕0.12	0.18∕0.18	Synergistic/synergistic
	Ampicillin	2/2	0.12/0.12	0.06∕0.06		

a *The MIC and MBC of cyclic dipeptides with ampicillin*.

b *The fractional inhibitory concentration index (FIC index)*.

c *The fractional fungicidal concentration index (FFC index)*.

### Combination of cyclic dipeptides and ampicillin significantly increases the inhibition of biofilms formation

**Figure 4:**
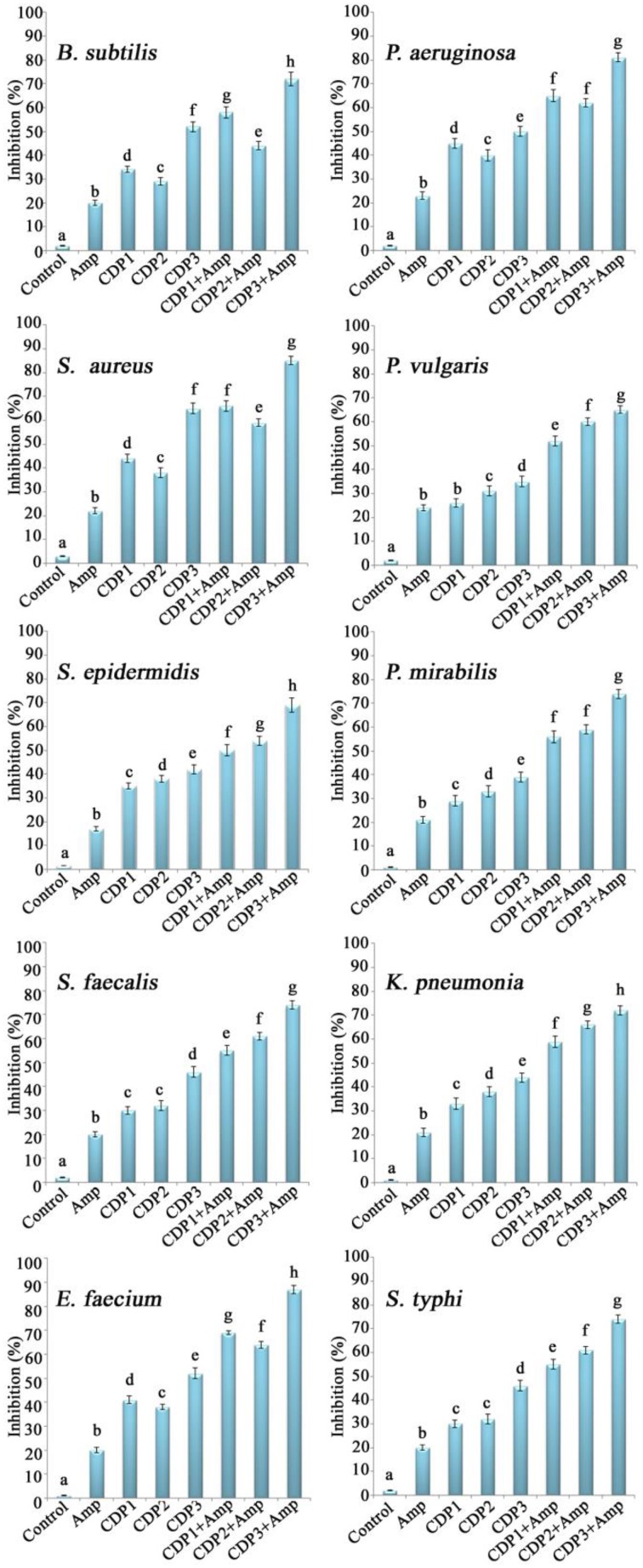
**Effects of the synergistic combination of cyclic dipeptides and ampicillin against biofilm formation by wound associated bacterial pathogens**. Error bars indicate the standard deviations of three measurements. Different letters in the superscript were significantly different according to Duncan's multiple range test (*p* < 0.05).

The effects of the combination of CDPs and ampicillin on the inhibition of wound associated bacterial biofilm formation were shown in Figure 4. The combination of CDPs and ampicillin recorded significant inhibition in the biofilm formation by the bacterial pathogens when compared to the effect of individual compounds. Significant inhibition in the biofilms was recorded by CDP 3 in combination with ampicillin when compared to the other CDPs.

### Immunomodulatory actions of cyclic dipeptides

**Figure 5:**
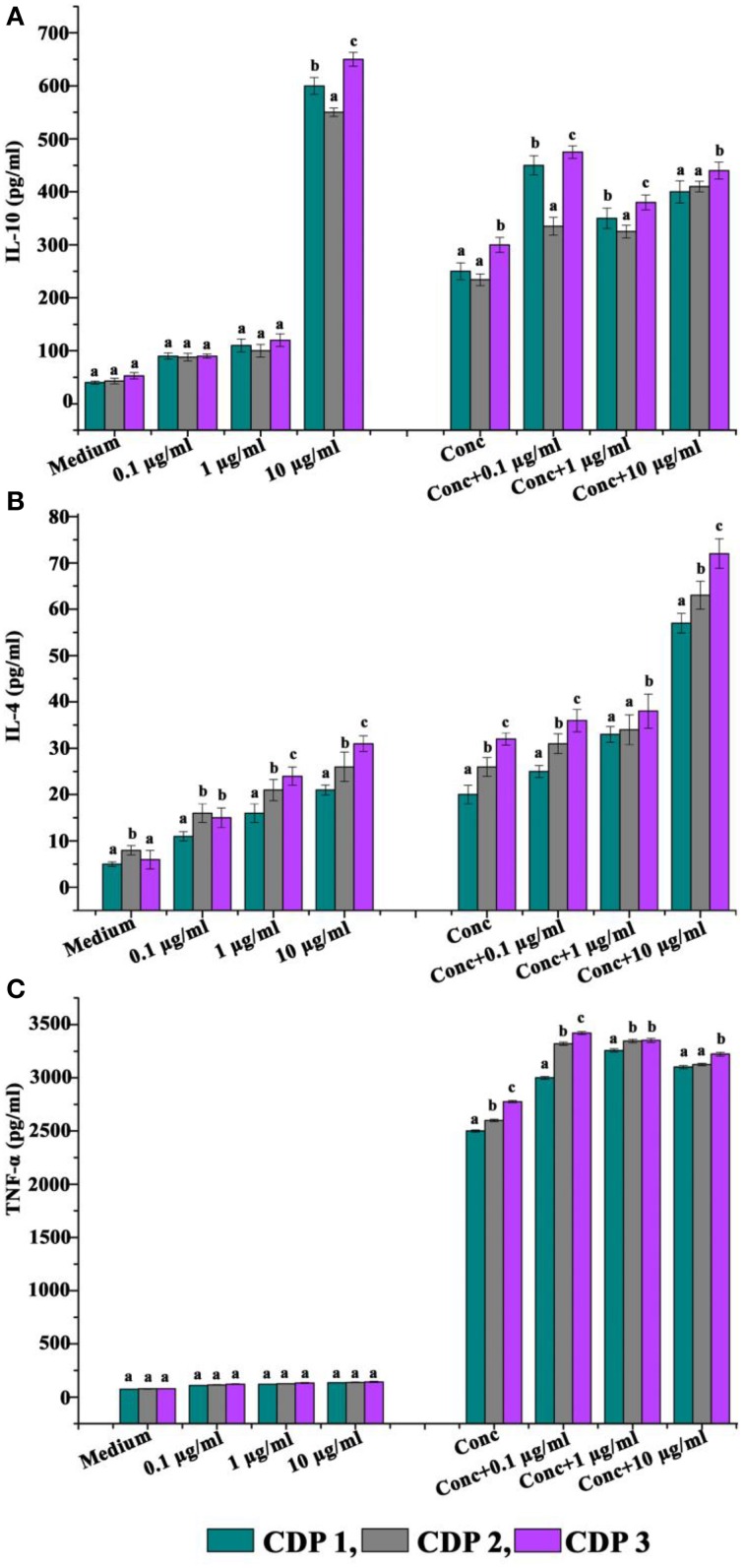
**Effects of cyclic dipeptides on the production of (A) IL-10, (B) IL-4, and (C) IFN-α by unstimulated and concanavalin A stimulated human peripheral blood mononuclear cells**. Error bars indicate the standard deviations of three measurements. Different letters in the superscript were significantly different according to Duncan's multiple range test (*p* < 0.05).

The abilities of CDPs to modulate the production of cytokines are shown in Figure 5. Incubation with the CDPs significantly enhanced the production of cytokine IL-10 by unstimulated cells and also boosted the production in cells stimulated with concanavalin A (standard T cell mitogen activator) (Figure 5A). Production of IL-4 by unstimulated cells was significantly enhanced in the presence of 10 μg/ml CDPs, but the compound was without significant effect on concanavalin A-stimulated cells (Figure 5B). Production of the pro-inflammatory cytokine TNF-α was not affected by incubation with the CDPs in the presence or absence of concanavalin A (Figure 5C).

### Cyclic dipeptides recorded significant toxicity toward the intracellular *S. aureus*

**Figure 6:**
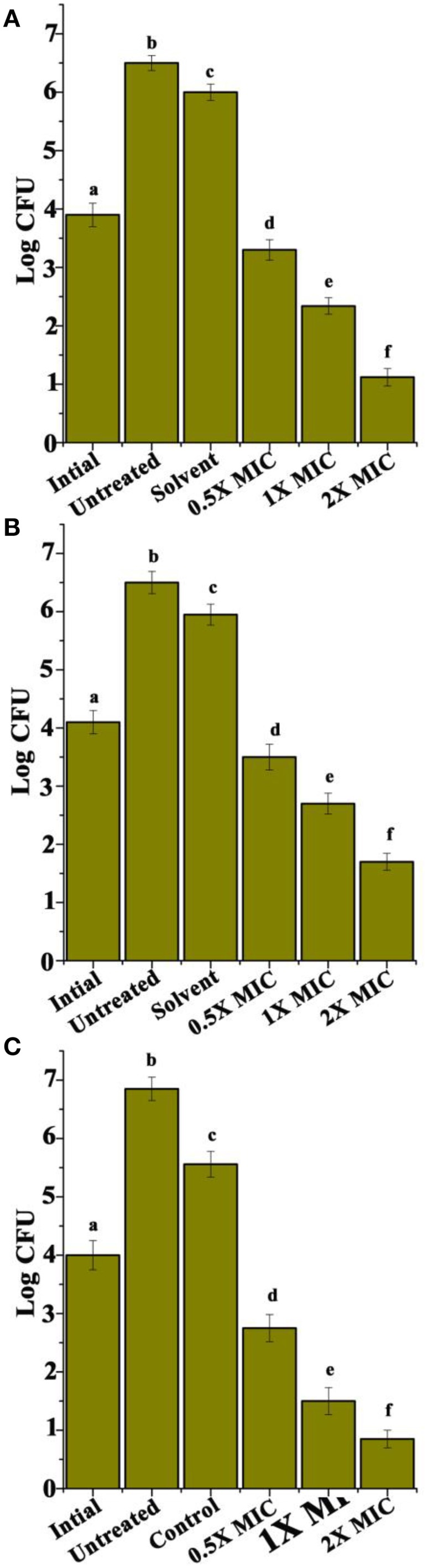
**Intracellular efficacy of rifampicin in J774 murine macrophage**. **(A)** CDP 1, **(B)** CDP 2, and **(C)** CDP 3. Experiments were performed three times. Error bars indicate the standard deviations of three measurements. Different letters in the superscript were significantly different according to Duncan's multiple range test (*p* < 0.05).

Infected murine J774 cells were treated with 1X and 2X MIC concentrations of CDPs. Significant decreases in bacterial loads were consistently observed for the treatment of CDPs (Figure 6), whereas none was observed with the solvent control (Figure 6). However, CDP 3 treatments significantly reduced the number of viable intracellular *S. aureus* at their respective 0.5X, 1X, and 2X MIC concentrations in J774 macrophages. Interestingly, cytotoxicity was not recorded for the uninfected macrophages by CDPs (data not shown).

### Cyclic dipeptides recorded no cytotoxicity toward normal cell lines

**Figure 7:**
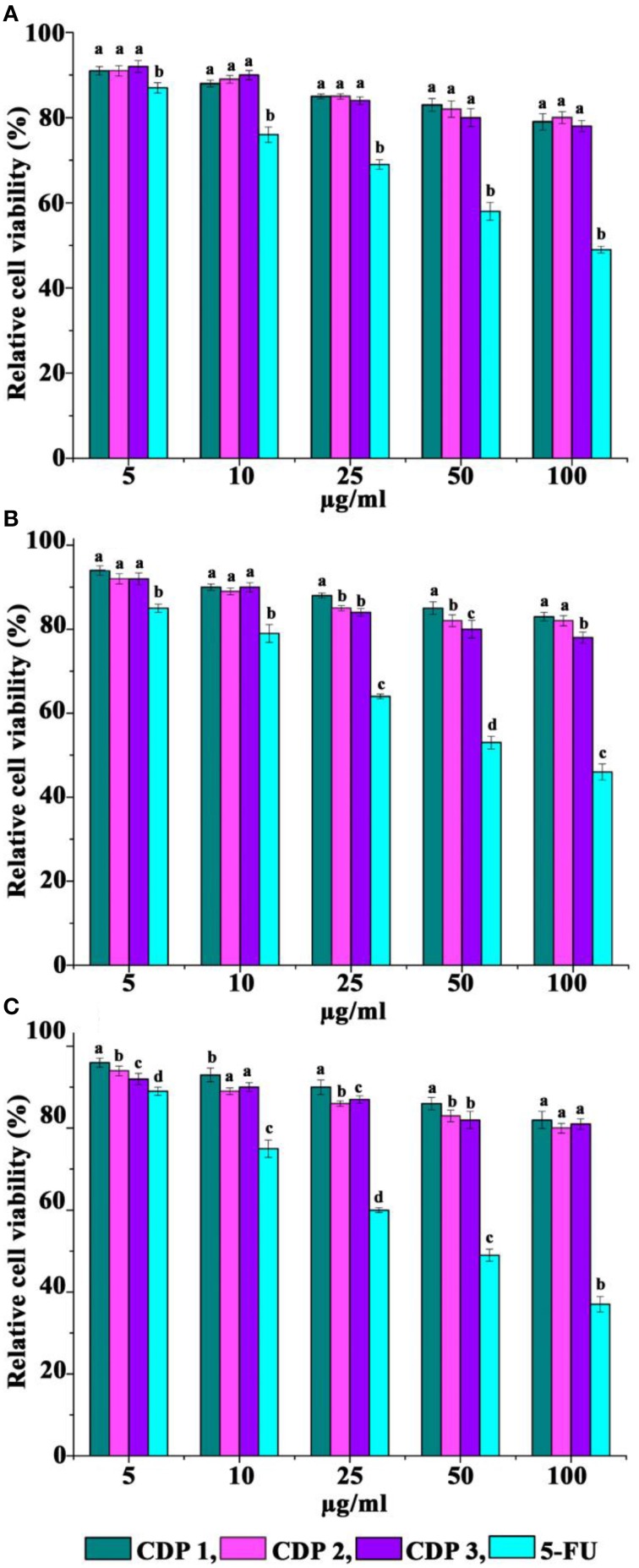
**Cytotoxicity of cyclic dipeptides against normal cell lines**. **(A)** FS normal fibroblast, **(B)** VERO, and **(C)** L231 normal lung epithelial. Error bars indicate the standard deviations of three measurements. Different letters in the superscript were significantly different according to Duncan's multiple range test (*p* < 0.05).

Cytotoxicity activity of CDPs against three normal cell lines after 72 h of treatment was recorded by MTT assay and was presented in Figure 7. When exposed to CDPs in the range, 5–100 μg/ml, CDPs recorded no cytotoxicity against FS, VERO, and L231 cell lines (Figure 7).

## Discussion

Cyclic dipeptides or CDPs (diketopiperazines or 2,5-Diketopiperazines or 2,5-DKP) are the smallest cyclic peptides known so far and are generally biosynthesized from various amino acids by a large variety of organisms, including microorganisms, sponges, mammals etc. (Sammes, 1975; de Carvalho and Abraham, 2012). The ability of various microorganisms including bacteria and fungi to synthesize CDPs is widespread and based on published reports more than 90% of Gram-negative bacteria produce them (de Carvalho and Abraham, 2012). CDPs have also been isolated from various Gram-positive bacteria (Stierle et al., 1988; De Rosa et al., 2003), fungi (Bugni and Ireland, 2004), and higher marine organisms including sponges (Huang et al., 2010). Significant biological activities of CDPs include anticancer, antiviral, antifungal, antibacterial, antiprion, antihyperglycemic, and glycosidase inhibitor agents etc. (de Carvalho and Abraham, 2012). Some CDPs recorded affinities for calcium channels and opioid, GABAergic, serotoninergic 5-HT1A and oxytocin receptors (de Carvalho and Abraham, 2012). Numerous studies are focused on important biological properties of CDPs connected to the inhibition of plasminogen activator inhibitor-1 (Folkes et al., 2001; Seko et al., 2002; de Carvalho and Abraham, 2012) and modification of cardiovascular and blood-clotting functions (McCleland et al., 2004; Cheng and Manwell, 2005). It is also well reported that some CDPs can block the development of physical dependence in mice (Walter et al., 1979; de Carvalho and Abraham, 2012). Moreover, CDPs are able to trigger quorum-sensing systems of bacteria via LuxR-mediation, and even they are reported to influence cell to cell communication in bacteria (Withers et al., 2001; Abraham, 2005; LaSarre and Federle, 2013). In addition, they have been reported as part of antibacterial nucleosides (Ichikawa and Matsuda, 2008).

CDPs are abound in nature and are often formed during the processing of various food items, degradation products of polypeptides, and beverages (Borthwick, 2012). They are usually formed during the condensation of two α-amino acids. According to Borthwick (2012) “CDPs occur in numerous natural products, and this subunit is often found alone or embedded in larger, more complex architectures in a variety of natural products from microbes, plant, and mammals.” CDPs have highly privileged structures that have the capability to bind to various biological receptors and also have several structural characteristics which make them attractive scaffolds for drug discovery programmes against various diseases and disorders (Borthwick, 2012). Moreover, CDPs are highly stable to proteolysis. CDPs are one of the crucial molecules in drug discovery programmes because its rigid backbone, which can mimic a preferential peptide conformation without any unwanted physical and metabolite properties of peptides (Borthwick, 2012). Borthwick (2012) observed that “the three-dimensional structure of CDPs can overcome one of the limitations of conventional medicinal agents, namely, the typical planarity found in most known pharmaceutical compounds discovered through the organic synthesis.” CDPs are easily synthesized from readily available α-amino acids and this can be used as core molecules around which we can construct combinatorial libraries of compounds (Borthwick, 2012). The advanced researches in solid-phase methodology have made CDPs even more attractive to combinatorial drug discovery programmes (Borthwick, 2012).

Previous literature reviews on CDPs focused on various natural chemicals comprising the 2,5-DKP ring (Borthwick, 2012). The source, chemical synthesis, biogenesis, and various physicochemical properties of CDPs were thoroughly studied in 1975, but the chemistry, biology, and pharmacological properties of many naturally occurring CDPs were reviewed only in 1990 (Borthwick, 2012). In 1993, the chemical synthesis, characteristics, chemical reactivity, and biological applications of CDPs were well-reported and many other beneficial bioactivities of simple cyclic peptides were reviewed in 1995 (Borthwick, 2012). In 2007, a short review focusing on the synthesis and biology were published, and very recently in 2012, a report exclusively based on properties, formation, and bioactivity of the monocyclic 2,5-DKP was reported (Borthwick, 2012).

Cyclo(L-Leu-L-Arg) has been previously reported as a natural compound from *Streptomyces* species (Tatsuta et al., 1972; Smaoui et al., 2011) or obtained by chemical synthesis (Sasaki et al., 1982). Till dated cyclo(D-Leu-D-Arg) is not reported from the natural source and is reported here for the first time from *Achromobacter* sp. Antibacterial activities of this compound against various Gram-positive and Gram-negative bacteria, as well as antifungal activities of cyclo(L-Leu-L-Arg), have also reported (Li et al., 2011). Smaoui et al. (2011) reported the antifungal activity of cyclo(L-Leu-L-Arg) only against *Fusarium oxysporum* by simple disc diffusion assay. In our previous study, we reported the antifungal activity of cyclo(L-Leu-D-Arg) isolated from *Bacillus* sp. N strain active against seven medically and agriculturally important fungi (Kumar et al., 2014c). But in the present study, the antibacterial activity of cyclo(D-Leu-D-Arg) against some clinically relevant wound pathogens is reported here for the first time. Chemical synthesis of stereoisomers of cyclo(Trp-Arg) is reported earlier (Li et al., 2012), but the isolation of cyclo(Trp-Arg) from natural source is not reported earlier in literature and to the best knowledge this is the first report on the isolation of cyclo(Trp-Arg) from natural source, especially from bacteria. Here, in the present study we reported the isolation of two enantiomers of cyclo(Trp-Arg), i.e., cyclo(L-Trp-L-Arg) and cyclo(D-Trp-D-Arg). In the present study cyclo(Trp-Arg) recorded excellent antibacterial activity against bacteria associated with wound infections and the best activity was recorded by cyclo(D-Trp-D-Arg). The antibacterial properties of cyclo(Trp-Arg) is reported here for the first time.

The CDPs are heterocyclic molecules which can exist in many stereoisomeric forms, and also in DD, LL, DL, and LD forms (Kumar et al., 2014d). The stereochemistries play an important role in the biological activity of CDPs. Antibacterial activities of DD- and DL-CDPs [cyclo(Pro-Val)] against *Vibrio anguillarum* were reported earlier, where cyclo(D-Pro-D-Val) recorded significantly better MIC values (0.05 μg/ml) than cyclo(D-Pro-L-Val) (0.11 μg/ml) (Fdhila et al., 2003; Kumar et al., 2014d). Huberman et al. (2007) reported that out of three CDPs enantiomers (LL, DL, and DD) tested, DD-enantiomer recorded best antimicrobial activity than the LL-enantiomer especially against *S. aureus* and *Micrococcus luteus*. Based on these results, the authors concluded that out of the three CDPs enantiomers tested and DD-enantiomers recorded enhanced bioactivity than their LL-forms. In the current investigation cyclo(D-Trp-D-Arg) recorded significant antibacterial activity than cyclo(L-Trp-L-Arg) and this may be due to the difference in the chirality properties of the tryptophan and arginine that make the change of target binding orientation of cyclo(Trp-Arg). The higher activity of cyclo(D-Trp-D-Arg) suggests that, it can form different spatial orientation that play a significant role in the enhancement of biological activity of this CDP. Stereochemistry of the CDPs play a vital role in the improvement of bioactivity has been reported previously on the inhibition of *Bacillus* sp. chitinase by cyclo(Arg-Pro), where cyclo(L-Arg-D-Pro) is more bioactive than cyclo(L-Arg-L-Pro) and this due to the existence of D-proline in active site (Houston et al., 2004). We also previously reported that, cyclo(D-Tyr-D-Phe) was more active than Cyclo(L-Tyr-L-Phe) (Kumar et al., 2014d). But linking other DD and LL CDPs reported in the literature, no strong connection between the enantiomers and their significant role in the enhancement of bioactivities can be elucidated. A systematic study on various CDPs and their corresponding enantiomers could reveal more information on the enhanced bioactive potential of simple CDPs (de Carvalho and Abraham, 2012).

Chronic wounds/burns are urgent global health problem affecting human population and bacterial infection plays a very significant role in these wounds to heal (Müller et al., 2013). Treatment of chronic wounds with diverse bacterial flora often involves synergistic combinations of drugs in an attempt to enhance efficacy and this will also reduce the emergence of antibiotic resistance (Müller et al., 2013). The synergistic effect of two antibiotics is defined as a greater-than-2-log increase in bactericidal activity *in vitro* compared with the bactericidal activity of each antibiotic alone (Müller et al., 2013). Here, we show conclusively that the combination of CDPs and the antibiotic ampicillin has a significant synergistic outcome on antibacterial activity against wound associated bacterial pathogens. The synergistic effect of CDPs with antibiotics is reported earlier (Kumar et al., 2013, 2014e). The synergistic effect of natural compounds and antibiotics are a thrust area of medicinal research with a vision for developing novel drug combination to fight against resistant microbes. Due to the ever increase prevalence of multi-drug resistant bacteria, synergistic testing using various combinations of microbial natural compounds with clinically used antibiotics could be a powerful tool in helping to select an antimicrobial chemotherapy more effective in low quantity, which may reduce the toxicity.

In infected chronic wound/burn, bacterial pathogens usually survive within protective structures often termed as biofilms (Bjarnsholt et al., 2008). Biofilms are often embedded in a glycocalyx (mixture of extracellular polysaccharides secreted by bacteria), that form a protective matrix adhering to the host's surrounding tissues around wounds/burns (Nidadavolu et al., 2012). Moreover, biofilms play a very significant role in the progression of many infectious diseases. Biofilms are also formed when planktonic bacteria adhere to a host's surface and initiate the formation of a microcolony that exists as a community enclosed in a protective extracellular polysaccharides secreted by bacteria. Moreover, biofilms account for more than 90% of wound/burn infections affecting humans and animals. In biofilms, bacteria display differential expression of many genes and are 1000-times more resistant to many antibiotics that are used for treatments (Huigens et al., 2007). Therefore, biofilms are highly resistant to host immune responses and various antibiotics used for treatments (Donlan, 2011) and failure to eliminate biofilms may eventually lead to persistent infections in the human body (Nidadavolu et al., 2012). Here, in the present study, CDPs and combination of CDPs with ampicillin reduces biofilm formation for various test Gram-positive and Gram negative bacteria. Thus, CDPs are promising candidate molecules in the search for a broad-spectrum biofilm inhibitor.

## Conclusion

Previously an enormous diversity of microbial bioactive compounds has been reported, but only a small portion of organisms (< 1%) in nature has been explored and much more can be expected in future. The structural variety of CDPs is very high and there are numerous recent reports available on new CDPs with diverse biological activities and this widens the variety of this group of compounds. The antibacterial activities of the CDPs especially against wound associated human pathogenic bacteria have never reported earlier. Here, we reported arginine based CDPs as a novel antibacterial agent against wound associated bacteria. Moreover, this compound recorded significant synergistic effect with ampicillin and also in inhibiting the biofilms formed by bacteria in *in vitro* conditions. Significant inhibition of biofilms was recorded by the combination of CDPs and ampicillin. Further, studies are also needed to determine the underlying modus operandi of CDPs on biofilm causing bacteria. Antibacterial activity of arginine based CDPs is an encouraging bioprobe to develop new antibacterial therapeutics in the near future especially as topical agents against many wound pathogens affecting human beings.

### Conflict of interest statement

The authors declare that the research was conducted in the absence of any commercial or financial relationships that could be construed as a potential conflict of interest.
